# Herpetic Keratitis and Corneal Endothelitis Following COVID-19 Vaccination: A Case Series

**DOI:** 10.7759/cureus.20967

**Published:** 2022-01-05

**Authors:** Hassan Alkwikbi, Mohammed Alenazi, Wafi Alanazi, Shahad Alruwaili

**Affiliations:** 1 Ophthalmology, King Saud Medical City, Riyadh, SAU; 2 Ophthalmology, Security Forces Hospital, Riyadh, SAU; 3 Ophthalmology, Alhammadi Hospital, Riyadh, SAU; 4 Ophthalmology, Imam Mohammed Ibn Saud Islamic University, Riyadh, SAU

**Keywords:** covid-19, case report series, covid-19 in ophthalmology, covid-19 vaccine complication, hsv-1

## Abstract

Herpetic corneal disease is the most common infectious cause of corneal blindness in developed countries. The majority of the infections are caused by the reactivation of the latent virus in the trigeminal ganglion. Environmental factors and physical stress are thought to contribute to viral reactivation. The pathognomonic lesion of the herpes simplex virus (HSV) is dendritic keratitis, which is visible on slit-lamp examination after fluorescein dye staining. A potential association between HSV reactivation and coronavirus disease 2019 (COVID-19) vaccines has been reported. In this case series, we present four cases of HSV reactivation in patients who received COVID-19 vaccination in Saudi Arabia from different medical centers. This report emphasizes the necessity of evaluating HSV reactivation as a potential side effect of COVID-19 vaccination. This is important because early diagnosis and timely management of herpetic lesions can potentially reduce the severity of infection.

## Introduction

Coronavirus disease 2019 (COVID-19) is a highly contagious viral infection caused by severe acute respiratory syndrome coronavirus 2 (SARS-CoV-2) that resulted in a global catastrophe and a significant loss of human life. The clinical spectrum of COVID-19 ranges from an asymptomatic stage to severe illness (acute respiratory distress and multiple organ failure). Fever (83%), cough (82%), and shortness of breath (31%) are the most common symptoms of SARS-CoV-2 infection [1]. Although the respiratory system is the primary target of SARS-CoV-2, it may cause cardiovascular, gastrointestinal, renal, hepatic, central nervous system, and ocular manifestations [2].

Although SARS-CoV-2 infects people of all ages, the elderly (>60 years), smokers, transplant patients, and individuals with comorbid conditions such as obesity, diabetes, cancer, cardiovascular diseases, chronic renal diseases, and chronic lung diseases are at a higher risk of developing severe COVID-19 infection [2]. The incidence of ocular symptoms in COVID-19 patients varies from 2% to 32%, manifesting at any stage of the disease. A study reported that the median duration between the appearance of COVID-19 and neuro-ophthalmic symptoms was five days, 8.5 days for ocular surface and anterior segment manifestations, and 12 days for the posterior segment and orbital manifestations [2].

It has been suggested that the virus can be transmitted through conjunctival inoculation, blood, or the upper respiratory tract to the nasolacrimal duct and lacrimal gland [4].

The ocular manifestations are divided into the following three categories based on the site of appearance: anterior segment, posterior segment, and neuro-ophthalmic. Anterior segment manifestations include conjunctivitis, which presents as tearing, conjunctival hyperemia, and foreign body sensation. Both unilateral and bilateral conjunctivitis, keratitis, and episcleritis have been reported in different studies [5-8]. Posterior segment manifestations involve the retina and optic nerve. These can be due to the involvement of the ganglionic cells, inner plexiform layer, optic disc, and retina [9,10]. Neuro-ophthalmic manifestations include cranial nerve palsies that can cause ophthalmoplegia [11,12].

Because of the global impact of the COVID-19 pandemic, vaccine development and production have been accelerated to contain the virus. Oxford-AstraZeneca and Pfizer-BioNTech are the two COVID-19 vaccines available in Saudi Arabia. According to the Centers for Disease Control and Prevention, the possible side effects of the vaccines include pain, swelling, redness at the injection site, headache, muscle pain, and fatigue.

Here, we report four cases of herpetic keratitis and corneal endothelitis following the completion of a two-dose COVID-19 vaccination (either Oxford-AstraZeneca or Pfizer-BioNTech).

## Case presentation

Case 1

An 18-year-old female reported to the ophthalmology clinic of a private hospital in May 2021 with complaints of pain, photophobia, and lacrimation from the right eye for over a month. The patient wore contact lenses and provided a history of swimming with the lenses. The patient had received two doses of the Pfizer-BioNTech COVID-19 vaccine and had developed symptoms a week after the second dose. The ocular examination revealed no abnormalities in the pupils, extraocular muscles, or visual field. The affected eye showed conjunctival hyperemia, normal intraocular pressure, no sub-tarsal foreign body, and hand motion vision. Fluorescein staining revealed the characteristic features of herpetic keratitis, including dendritic corneal ulcers, and the posterior cornea showed precipitates. The anterior chamber of the right eye was deep and showed a mild cellular reaction. The retinal examination of both eyes and the left eye slit-lamp examination were unremarkable. The patient was diagnosed with herpetic keratitis and was treated with ganciclovir ophthalmic gel 0.15% five times daily eye drops and lubricants. Although the patient showed improvement during subsequent visits, the epithelial inflammation persisted. She was referred to a tertiary care facility to rule out acanthamoeba keratitis.

Case 2 

**Figure 1:**
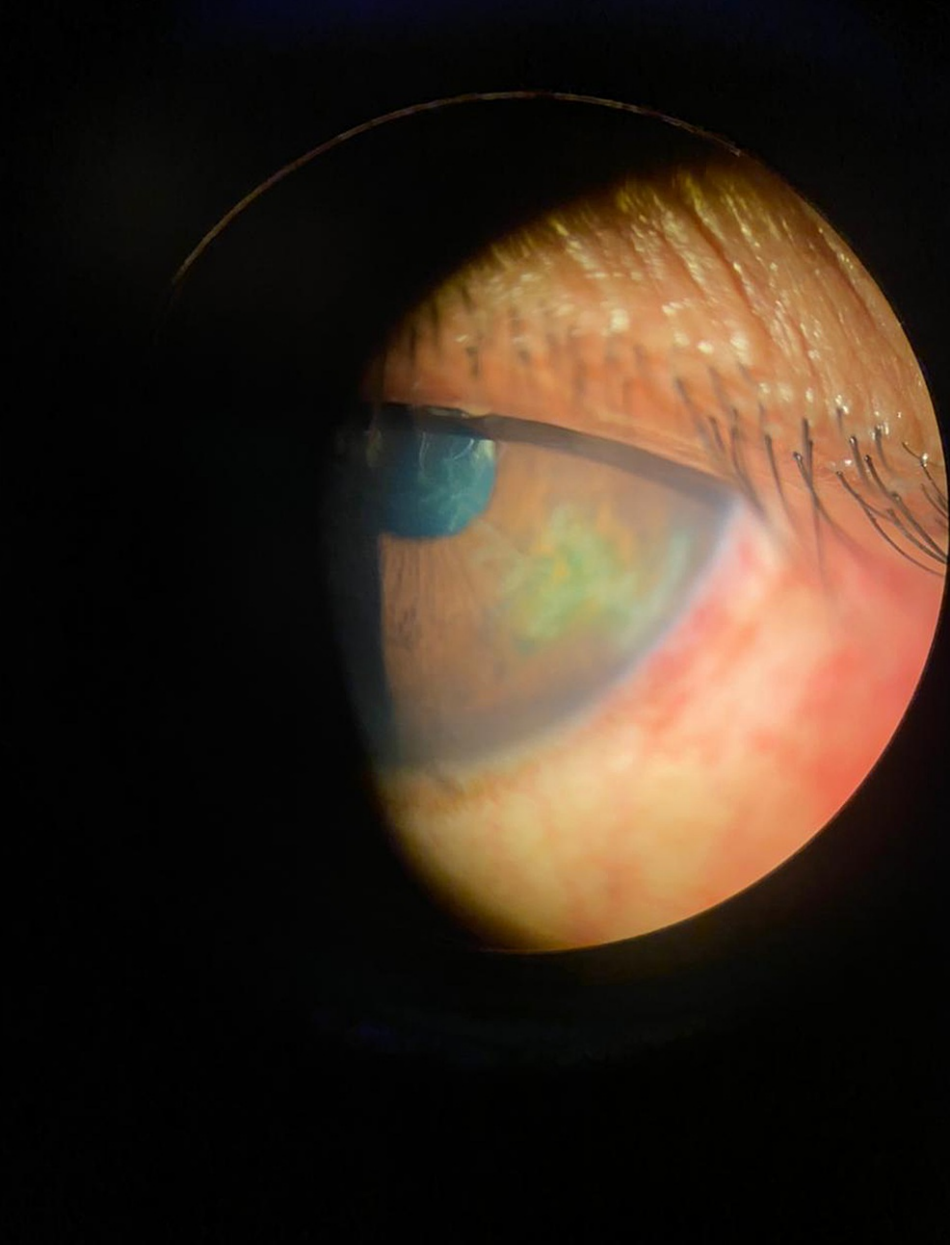
Slit-lamp examination showing dendritic corneal ulcer after COVID-19 vaccination. COVID-19: coronavirus disease 2019

**Figure 2:**
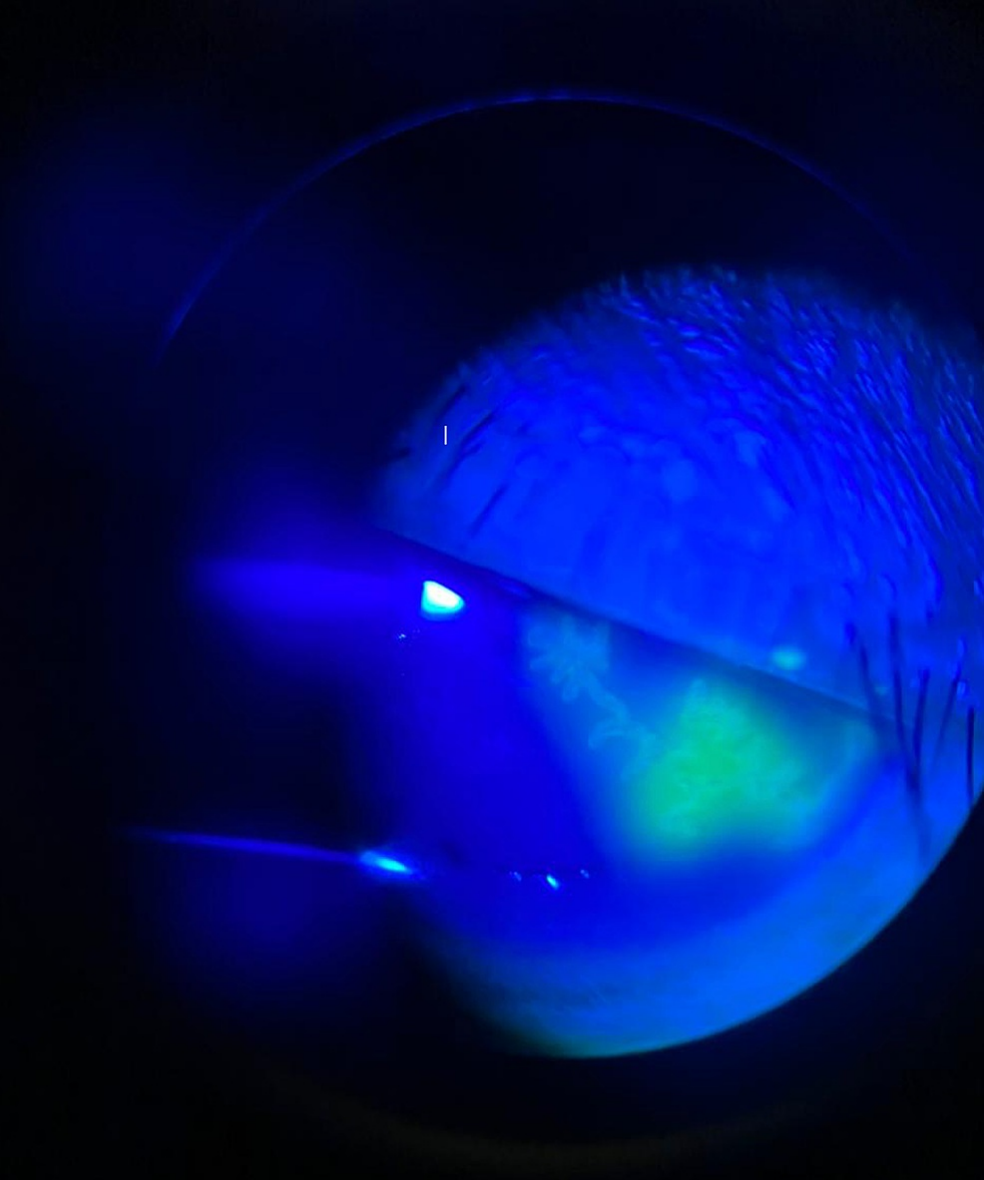
The dendritic corneal ulcer stained with fluorescein.

A 40-year-old Indian male presented to the ophthalmology clinic with complaints of pain, tearing, and photophobia in the right eye. His ocular, medical, and family histories were unremarkable. He reported no known allergies. He had received two doses of the Pfizer-BioNTech COVID-19 vaccine and had developed ocular symptoms a week after the second dose. The visual acuities of the right and left eyes were 25/20 and 20/20, respectively. The pupils, extraocular muscles, and confrontation fields were normal. The left anterior segment was also clear. However, the right anterior segment showed a typical dendritic ulcer of the peripheral cornea (Figure 1). Fluorescein staining revealed dendritic epithelial keratitis (Figure 2). Applanation tonometry was used to measure the intraocular pressure (20 mmHg). The patient was diagnosed with herpetic keratitis. He was prescribed lubrication, ganciclovir ophthalmic gel 0.15% five times daily, and oral acyclovir 400 mg five times daily for 10 days. He was scheduled to return to work in three weeks.

Case 3

A 32-year-old male presented to the ophthalmology clinic with chief complaints of pain, redness, and blurry vision in the right eye. The patient provided no significant family or medical history (rheumatic disease, tumors, trauma). The patient had received the second dose of the Oxford-AstraZeneca vaccine a week before the onset of symptoms. On examination, the best-corrected visual acuity was 6/20 in the right eye and 20/20 in the left eye. The intraocular pressure in the right eye was 40 mmHg. A slit-lamp examination showed corneal edema and ciliary congestion. Epithelial dendrites were noted on the surface of the cornea, and the anterior chamber of the right eye was shallow. The patient was diagnosed with herpetic keratouveitis glaucoma. He was prescribed prednisone at 1.0 mg/kg/day for four weeks, with gradual tapering according to the patient’s response and cyclopentolate 1% t.i.d.

Case 4

A 29-year-old male presented with pain, tearing, and redness in the left eye. He had undergone laser-assisted in-situ keratomileusis five years ago. His medication history was insignificant, no allergies were reported, and there was no history of herpes simplex virus (HSV). The patient had received the second dose of the Pfizer-BioNTech COVID-19 vaccine a week before the onset of the ocular symptoms. On examination, the visual acuity of both eyes was 20/20. A slit-lamp evaluation revealed stromal infiltration and diffuse conjunctival injection with a dendritic corneal ulcer. An anterior chamber trace was also noted. The patient was diagnosed with herpetic keratitis and was prescribed ganciclovir ophthalmic gel 0.15% five times daily and oral acyclovir 400 mg five times daily for 10 days. He was kept under observation and showed a remarkable improvement in the ocular symptoms.

## Discussion

Globally, herpetic keratitis is one of the leading causes of blindness. HSV-1 manifests as epithelial and stromal keratitis, conjunctivitis, herpetic blepharitis, and uveitis [13,14]. HSV transmission occurs through direct contact with body fluids (saliva or genital secretions). In addition, it can be transmitted through active oral lesions [15].

After primary infection, the HSV remains latent in the dorsal root or cranial nerve ganglia. Reactivation of the latent varicella-zoster virus (VZV) may be triggered by environmental stressors, corneal injury, fever, immunosuppression, and laser phototherapeutic keratectomy [16]. On reactivation, the virus may travel through the ophthalmic branch of the trigeminal nerve and infect the corneal basal epithelium and the ocular surface. HSV reactivation is often asymptomatic. However, ocular (epithelial keratitis, conjunctivitis) and cutaneous manifestations may occur in some cases. The ocular symptoms include pain, redness, discharge, blurred vision, and photophobia. A study reported two cases of herpetic keratits after the COVID-19 vaccine and found that the median duration between the COVID-19 vaccination and the onset of HSV was a couple of days after the first dose in one patient and after the second dose in another patient [17]. In our study, HSV reactivation occurred within one week following the second dose of the COVID-19 vaccine. In contrast, Alkhalifah et al. [18] reported two cases of HSV reactivation between four days and four weeks after receiving the vaccine. Recurrent HSV keratitis is associated with corneal scarring and astigmatism and may lead to blindness. The susceptibility of the host to ocular HSV infections depends on the status of the immune system. Various inherited and acquired diseases and the age of the affected individuals may limit the immune system’s effectiveness in preventing the recurrence of ocular HSV [15].

Reactivation occurs when the virus escapes the immune system’s ability to prevent it from replicating. However, the specific processes that cause herpes virus infection to reactivate after an inactivated COVID-19 vaccination remain unknown in our cases.

According to Ewer et al. [19], COVID-19 vaccination can trigger cytokine release and immune response cascades. This can result in the up-regulation of natural killer group 2D ligands, the reactivation of HSV or VZV from the latent phase, and the development of clinical HSV signs and symptoms following vaccination.

## Conclusions

This report emphasizes the necessity of evaluating HSV reactivation as a potential complication of COVID-19 vaccination. The COVID-19 pandemic is a global catastrophe and vaccination is imperative for reducing the transmission of SARS-CoV-2. Ophthalmologists must be cognizant of the potential reactivation of HSV following COVID-19 vaccination for the early diagnosis and timely management of the ophthalmic manifestations. Moreover, healthcare professionals must record the relevant history and be mindful of the clinical signs, particularly in vaccinated individuals with a history of HSV infection.
